# Comparison of actionable alterations in cancers with kinase fusion, mutation, and copy number alteration

**DOI:** 10.1371/journal.pone.0305025

**Published:** 2025-01-23

**Authors:** Shinsuke Suzuki, Toshiaki Akahane, Akihide Tanimoto, Michiyo Higashi, Ikumi Kitazono, Mari Kirishima, Masakazu Nishigaki, Toshiro Ikeda, Shuichi Kanemitsu, Junichi Nakazawa, Erina Akahane, Hiroshi Nishihara, Kimiharu Uozumi, Makoto Yoshimitsu, Kenji Ishitsuka, Shin-ichi Ueno

**Affiliations:** 1 Cancer Center, Kagoshima University Hospital, Kagoshima, Japan; 2 Department of Clinical Oncology, Course of Advanced Therapeutics, Kagoshima University Graduate School of Medical and Dental Sciences, Kagoshima, Japan; 3 Department of Hematology and Rheumatology, Kagoshima University Hospital, Kagoshima, Japan; 4 Department of Pathology, Kagoshima University Graduate School of Medical and Dental Sciences, Kagoshima, Japan; 5 International University of Health and Welfare (IUHW), Tokyo, Japan; 6 Department of Genetic Counseling, Kagoshima University Hospital, Kagoshima, Japan; 7 Department of Plastic Surgery, Sagara Hospital, Kagoshima, Japan; 8 Department of Medical Oncology, Kagoshima City Hospital, Kagoshima, Japan; 9 Keio Cancer Center, Keio University School of Medicine, Tokyo, Japan; 10 Department of Medical Oncology, National Hospital Organization Kagoshima Medical Center, Kagoshima, Japan; Osmania University, Hyderabad, India, INDIA

## Abstract

Kinase-related gene fusion and point mutations play pivotal roles as drivers in cancer, necessitating optimized, targeted therapy against these alterations. The efficacy of molecularly targeted therapeutics varies depending on the specific alteration, with great success reported for such therapeutics in the treatment of cancer with kinase fusion proteins. However, the involvement of actionable alterations in solid tumors, especially regarding kinase fusions, remains unclear. Therefore, in this study, we aimed to compare the number of actionable alterations in patients with tyrosine or serine/threonine kinase domain fusions, mutations, and copy number alterations (CNAs). We analyzed 613 patients with 40 solid cancer types who visited our division between June 2020 and April 2024. Furthermore, to detect alterations involving multiple-fusion calling, we performed comprehensive genomic sequencing using FoundationOne^®^ companion diagnostic (F1CDx) and FoundationOne^®^ Liquid companion diagnostic (F1LCDx). Patient characteristics and genomic profiles were analyzed to assess the frequency and distribution of actionable alterations across different cancer types. Notably, 44 of the 613 patients had fusions involving kinases, transcriptional regulators, or tumor suppressors. F1CDx and F1LCDx detected 13 cases with kinase-domain fusions. We identified 117 patients with kinase-domain mutations and 58 with kinase-domain CNAs. The number of actionable alterations in patients with kinase-domain fusion, mutation, or CNA (median [interquartile range; IQR]) was 2 (1–3), 5 (3–7), and 6 (4–8), respectively. Patients with kinase fusion had significantly fewer actionable alterations than those with kinase-domain mutations and CNAs. However, those with fusion involving tumor suppressors tended to have more actionable alterations (median [IQR]; 4 [2–9]). Cancers with kinase fusions exhibited fewer actionable alterations than those with kinase mutations and CNAs. These findings underscore the importance of detecting kinase alterations and indicate the pivotal role of kinase fusions as strong drivers of cancer development, highlighting their potential as prime targets for molecular therapeutics.

## Introduction

The ability to determine complete exon sequencing of relevant tumor drivers, suppressors, and resistance genes is paramount for optimizing personalized medicine. Comprehensive genome sequencing (CGS) is a valuable tool for detecting actionable alterations, which provide the biological basis of tumorigenesis. Kinase-related gene fusions, point mutations, and copy number alterations (CNAs) are critical drivers in cancer, necessitating optimized targeted therapy against these alterations. The FoundationOne^®^ companion diagnostic (F1CDx) and FoundationOne^®^ Liquid companion diagnostic (F1LCDx) are tissue- and blood-based broad companion diagnostics approved by the Food and Drug Administration and clinically and analytically validated for all solid tumors. In Japan, F1CDx and F1LCDx are covered by public health insurance only after completion of standard treatment, which differs from practices in other countries [1].

Gene fusion, particularly that involving tyrosine kinase, is pivotal as a driver mutation in cancer development. A hallmark example is the Philadelphia chromosome 9–22 translocation, characteristic of chronic myelogenous leukemia, which generates the fusion protein BCR–ABL1 [2, 3]. Chronic myelogenous leukemia, driven by the tyrosine kinase gene fusion *BCR–ABL1*, stands out as the tumor with the most notable success in molecularly targeted therapeutics to date. Notably, approximately 11 years of follow-up have shown that the efficacy of imatinib, a molecularly targeted drug, persists over time [4].

The prognosis of patients with non-small cell lung cancer (NSCLC) harboring oncogenic driver-gene alterations, such as epidermal growth factor receptor (*EGFR*) kinase mutation, anaplastic lymphoma kinase (*ALK*) fusion, c-ros oncogene 1 (*ROS1*) fusion, or rearranged during transfection (*RET*) fusion, those with pan-cancer harboring neurotrophic tyrosine receptor kinase genes 1/2/3 fusion, and those with cholangiocarcinoma harboring fibroblast growth factor receptor 2 (*FGFR2*) fusion has improved prominently with the introduction of molecularly targeted drugs. However, the therapeutic effects of tyrosine kinase inhibitors (TKIs) vary between *EGFR* mutation and *ALK*, *ROS1*, *RET*, and neurotrophic tyrosine receptor kinase genes 1/2/3 *FGFR2* fusion. A tail-plateau, synonymous with a durable response on a Kaplan–Meier survival curve, represents an attractive effect of molecularly targeted therapies [4] and immune checkpoint inhibitors [5, 6] and has great clinical significance. In patients with advanced NSCLC with *EFGR* mutations, the first generation of gefitinib and the third generation of osimertinib, which is effective against the resistance mutation EGFR T790M, limited the durability of the response and did not show the benefit of tail-plateau on progression-free survival curves [7, 8]. However, TKIs in NSCLC cases with *ALK* [9, 10], *ROS1* [11], and *RET* fusion [12] and in pan-cancer with neurotrophic tyrosine receptor kinase genes 1/2/3 fusion [13] exhibited a tail plateau on the progression-free survival curve. Furthermore, Gao et al. analyzed a cohort of 9,624 samples from The Cancer Genome Atlas with 33 cancer types to detect gene fusion events. They focused on kinase fusion and showed that tumors with fusion events tend to have a lower mutational burden [14].

Therefore, in this study, we hypothesized that the smaller the number of actionable alterations, the stronger the tumorigenesis, and that the efficacy of targeted therapy would depend on the driver kinase alterations. Thus, we aimed to compare the number of actionable alterations in patients with tyrosine or serine/threonine domain fusions, mutations, and CNAs using tissue- and blood-based CGS.

## Materials and methods

### Patients

In total, 613 patients with 44 different solid cancer types visited our division between June 2020 and May 2024. Of these, 504 (82.2%) and 109 (17.8%) patients were analyzed using tissue- and blood-based CGS, respectively. Furthermore, all patients had solid cancer with advanced stage, with a performance status of 0 or 1, and had completed standard treatment covered by Japanese health insurance. This cohort also included patients with untreated rare cancers and sarcomas lacking standard treatment protocols. We did not exclude patients tested by these assays during the data collection period, except those who were analyzed using F1LCDx and received anti-EGFR antibody therapy. This exception was due to blood-based sequencing identifying multiple novel mutations, copy gains, and fusions associated with anti-EGFR therapy that frequently co-occur as subclonal alterations in the same patient [15]. The 40 types of cancers that were included in this study were adenoid cystic carcinoma, bladder urothelial carcinoma, brain low-grade glioma, breast invasive carcinoma, cervical squamous cell carcinoma, endocervical adenocarcinoma, cholangiocarcinoma, colon adenocarcinoma, esophageal carcinoma, glioblastoma multiforme, head and neck squamous cell carcinoma, kidney renal clear cell carcinoma, kidney renal papillary cell carcinoma, lung adenocarcinoma, lung squamous cell carcinoma, mesothelioma, neuroendocrine tumor, NUT carcinoma, ovarian serous cystadenocarcinoma, pancreatic adenocarcinoma, pheochromocytoma and paraganglioma, porocarcinoma, salivary duct carcinoma, gastrointestinal stromal tumor, prostate adenocarcinoma, sarcoma, skin cutaneous melanoma, stomach adenocarcinoma, testicular germ cell tumor, thymoma, thyroid carcinoma, uterine carcinosarcoma, uterine corpus endometrial carcinoma, phyllodes tumor, cutaneous squamous cell carcinoma, appendiceal adenocarcinoma, Paget’s disease, and uveal melanoma. Patient characteristics, such as sex, age, smoking, heavy drinking (approximately ≥60 g of pure alcohol per day average), prior chemotherapy, previous targeted therapy, and F1CDx or F1LCDx analyses were postulated to affect the number of actionable alterations (Table 1).

**Table 1:** Baseline characteristics of different subgroups (n = 197).

	Kinase alteration			Fusion tumor suppressor	p-value
Factor	Fusion	Mutation	CNA		
N	13	117	58	9	
Male/Female, no. (%)	8/5 (61.5/38.5)	53/64 (45.3/54.7)	33/25 (56.9/43.1)	7/2 (77.8/22.2)	0.14
Median age (SD)	56.46 (19.21)	61.22 (13.92)	59.34 (13.11)	67.78 (12.14)	0.245
Never smoked (%)	7 (53.8)	62 (53.0)	25 (43.1)	4 (44.4)	0.636
Heavy drinking (%)	1 (7.7)	17 (14.5)	10 (17.2)	2 (22.2)	0.768
Without prior chemotherapy (%)	8 (61.5)	75 (64.1)	43 (74.1)	7 (77.8)	0.489
Without prior targeted therapy (%)	12 (92.3)	104 (88.9)	55 (94.8)	9 (100.0)	0.448
Blood-based CGS (%)	3 (23.1)	13 (11.1)	4 (6.9)	2 (22.2)	0.192
Median no. (IQR) of AA	2 (1–3)	5 (3–7)	6 (4–8)	4 (2–9)	<0.001

CGS, comprehensive genome sequencing; IQR, interquartile range; AA, Actionable alterations.

### Ethics approval

This study was conducted according to the Declaration of Helsinki and was approved by the Ethics Committee and Institutional Review Board of Kagoshima University (approval number 180053) (E-mail: isgskkrs@kuas.kagoshima-u.ac.jp). All patients provided written informed consent.

### Sequencing

Next-generation sequencing was performed using F1CDx and F1LCDx, CGS approaches involving the hybrid capture method. These tests targeted a panel of 324 genes, identifying base substitutions, insertions, deletion mutations, and copy number alterations in 309 genes; gene fusion in 36 genes; and tumor mutational burden (TMB), a measure of the number of somatic protein-coding base substitutions and insertion/deletion mutations, in a tumor specimen. Protein tyrosine kinase and serine/threonine kinase were analyzed for kinase-activating mutations. CNA of CDK4/6, which coexists with other kinase alterations, was excluded from the kinase CNA in this study. SS18-SSX and NAB2-STAT6 are disease-specific gene-fusion abnormalities detected separately through reverse-transcription polymerase chain reaction.

### Actionable alterations

Notably, all the detected gene alterations in cancer-related genes were annotated and curated using the COSMIC (https://cancer.sanger.ac.uk/cosmic), ClinVar (https://www.ncbi.nlm.nih.gov/clinvar/), CIViC (https://civicdb.org/home), SnpEff (https://www.accessdata.fda.gov/cdrh_docs/pdf17/P170019B.pdf.), and Clinical Knowledgebase (CKB) (https://ckb.jax.org/) databases. We calculated the validation (database; score) for mutation or fusion and the validation (score) for CNA or the clone status using PleSSision (Mitsubishi Space Software Co., Ltd., Tokyo, Japan), an outsourcing clinical sequencing system [10]. Mutations with a score of ≥2 points were considered actionable alterations. We used the following score tables:

- for mutation or fusion of oncogenes: well-known driver status (> 100 reports in COSMIC or pathogenic in ClinVar; 2), gain of function (CKB; 2), likely gain of function (CKB; 1.5), computational prediction of damage (SnpEff; 0.5);
- for mutation or fusion of tumor suppressor gene (TSG): germline loss of function (gLOF) (pathogenic in ClinVar; 2), gLOF (truncate mutation; 2), gLOF (CKB or likely pathogenic in ClinVar; 2), gLOF of computational prediction (SnpEff; 0.5), somatic loss of function (sLOF) (pathogenic in ClinVar; 1), sLOF (truncate mutation; 1), sLOF (CKB or likely pathogenic in ClinVar; 1), computational prediction of damage (SnpEff; 0.5);
- for CNA of an oncogene: neutral (0), copy number (CN)<4 (0), CN ≥4 (1), CN ≥8 (2),
- for TSG deletion, neutral (0), loss of heterozygosity (LOH) (1), uniparental disomy (UPD) (1), homologous deletion (HD) (2);
- for clone status: main clone (1), subclone, tumor content >50% (1), subclone (0), uncertain (0), not a cancer clone or inconsistent with pathology (0), not inherited by cancer clones (-0.5).

For example, the TP53 R282W (LOH) mutation is annotated as sLOF (pathogenic in ClinVar; 1), LOH (1), and main clone (1), with a cumulative score of 3. This would be considered an actionable alteration as per our criteria.

Furthermore, a multidisciplinary team comprising medical oncologists, pathologists, clinical laboratory technologists, bioinformaticians, and clinical geneticists conducted a comprehensive analysis to ascertain the clinical significance of these gene alterations. We referred to various databases and used an outsourcing clinical sequencing system to eliminate false negative actionable alterations.

### Statistical analysis

For the number of actionable alterations, data are presented as median and interquartile range. Statistical significance was determined using analysis of covariance and the Steel–Dwass multiple comparison test, and statistical significance was set at *P* < 0.05.

## Results

### Detection of fusion genes in kinases, transcriptional regulators, and tumor suppressors

**Fig 1:**
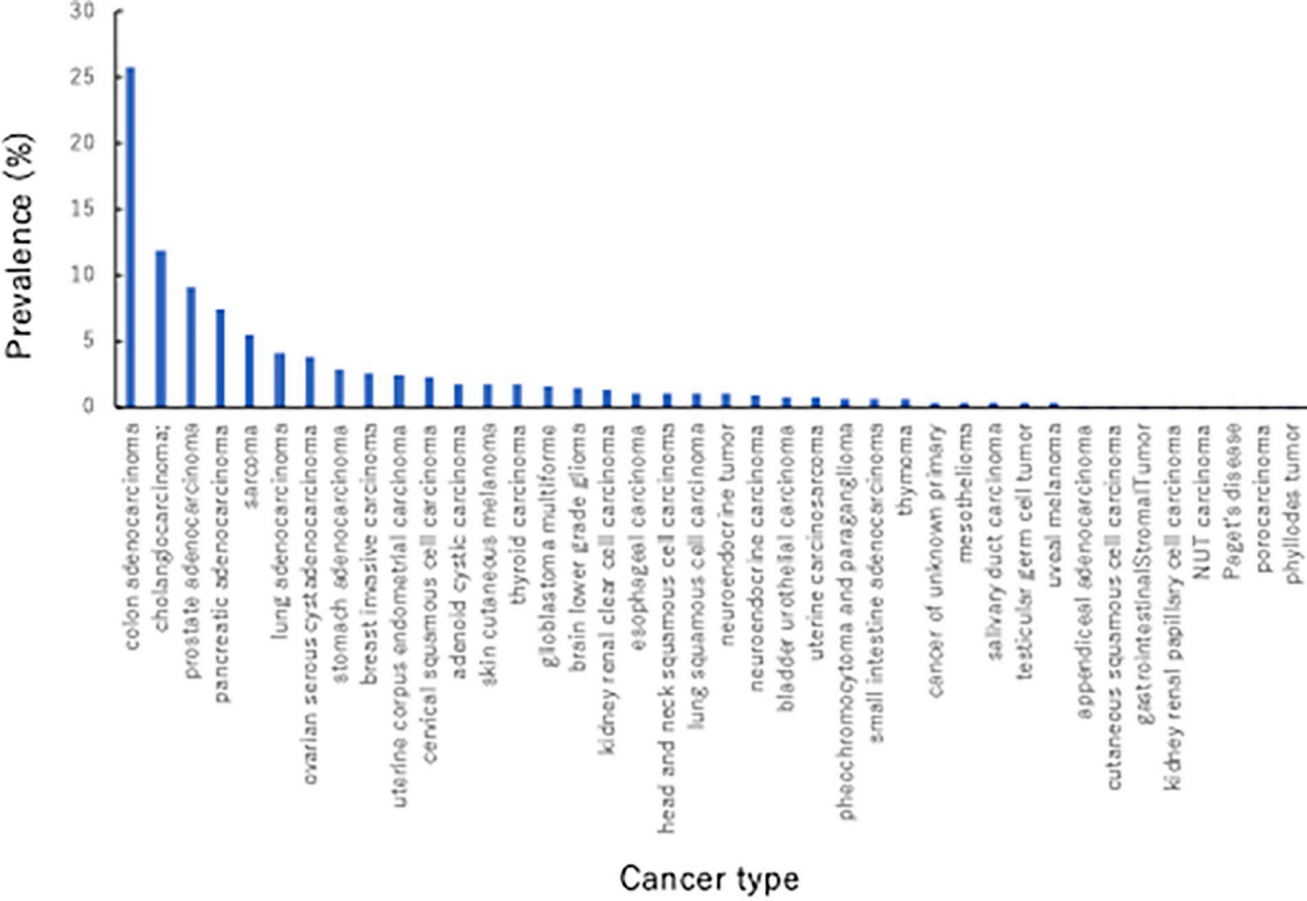
Proportions of cancer types in our sample. We analyzed the data of 613 patients with 40 different types of solid cancer using c tissue- and blood-based CGS.

We analyzed 613 patients with 40 solid cancer types via CGS. Fig 1 presents the distribution of patients with each cancer type. The most frequently encountered cancer types were colorectal cancer (25.8%), cholangiocarcinoma (11.9%), and prostate cancer (9.1%); however, we analyzed several cancer types ranging from major cancers to sarcomas (5.5%) and rare cancers (7.8%). We identified 44 patients (9.6%) with fusions involving kinases, transcriptional regulators, or tumor suppressors. F1CDx and F1LCDx helped to detect 13 patients (2.1%) with kinase-domain fusion (Table 2). Notably, in 12 of the 13 patients with kinase-domain fusion, except for patient 9 with *CDK6* CNA (CN = 98), the other kinase-domain mutations and CNAs were mutually exclusive (Tables 1 and 3). In addition, five of the 13 patients with kinase-domain mutations had cholangiocarcinoma (Tables 1 and 3). Patient 13, with an *FGFR2-TACC2* fusion, exhibited coexistence with serine/threonine kinase mutation, *MAP3K13 p*.*I523V*(LOH), which was categorized as a variant of unknown significance. Additionally, patients 5, 6, and 11 showed coexistence of *MYC* CNA (CN = 9), *KRAS* p.*Q61H*, and *MYC* CNA (CN = 11), respectively, which is an interpreted oncogene (Table 3). Notably, all 13 patients with kinase domain fusion revealed tyrosine kinase gene and were mutually exclusive of other tyrosine kinase mutations (Table 3). Furthermore, except for one case that could not be analyzed, remaining 12 cases demonstrated low TMB (Table 3). Although lung adenocarcinoma is associated with a high frequency of kinase fusions [9–12], no kinase fusion was observed in our analysis. This is due to the small number of patients with lung adenocarcinoma (4.1%) analyzed in this study (Fig 1).

**Table 2:** Fusion detection in cancer (n = 613).

Cancer type	Type, No. (%)	Fusion, 46 (7.5)		
		Kinase, 13 (2.1)	TR, 24 (3.9)	TS, 9 (1.5)
Adenoid cystic carcinoma		*-*	*MYB-NFIB*, *MYB-AHI1*	*-*
Breast invasive carcinoma		*-*	*-*	*PLAT-ETV6*
Cholangiocarcinoma		*TRIM4-MET* *ANKRD55-FGFR1* *MYH9-ALK* *FGFR2-BICC1 (2)*	*CALR-ZNF14*	*NF1-SSH2*, *TET2-UTRN*, *ATM-SLC35F2*
Colon adenocarcinoma		*SUPT3H-ROS1*,	*-*	*NF1-TANC2*
Glioblastoma multiforme		*FGFR1-TACC1*	*-*	*-*
Kidney renal clear cell carcinoma		*-*	*Xp11-TFE3*	*-*
Low-grade glioma		*KIAA1549-BRAF*	*C11orf95-RELA*, *MN1-BEND2*	*-*
Neuroendocrine tumor		*SORCS1-RET*	*MLL-PHC2*	*-*
NUT carcinoma		*-*	*NUTM1-BRD4*	*-*
Pancreatic adenocarcinoma		*PPP1R9A-BRAF*	*-*	*TTC39B-CDKN2A*
Porocarcinoma		*-*	*NUTM1-BRD4*	*-*
Prostate adenocarcinoma		*SPINT1-IGF1R*	*TMPRSS2-ERG (5)*, *ASXL1-STX16*	*-*
Sarcoma		*-*	SS18-SSX*, *TMPRSS2-ZNF317*, *ASPCR1-TFE3*, *MYOM1-STAT3*, *ATF1-EWSR1*, *EWSR1-FLI1(2)*, *EWSR1-NR4A3*, NAB2-STAT6*	*-*
Skin cutaneous Melanoma		*SUGT1P1-FGFR1*	-	*PTPN1-TMPO*
Stomach adenocarcinoma		*FGFR2-TACC2*	-	*CH1-CEBPA*, *POLE-ANKLE2*

TR, Transcriptional regulator; TS, Tumor suppressor.

Cancer types without fusions detected are not shown. Fusion genes detected multiple times are indicated by numbers in parentheses.

*Disease-specific gene-fusion abnormalities were detected separately through a reverse-transcription polymerase chain reaction.

**Table 3:** Actionable alterations of patients with kinase fusion (n = 15).

Patient	Cancer type	Kinase fusion	AA	No. of AA	TMB
1	Cholangiocarcinoma	*TRIM4-MET*	*-*	0	low*
2	Cholangiocarcinoma	*ANKRD55-FGFR1*	*IDH1 p*.*R132L*, *IKZF1 p*.R208*	2	low
3	Cholangiocarcinoma	*MYH9-ALK*	*APC p*.K139fs*30(LOH), *GATA6 CNA* (CN = 14)	2	low
4	Cholangiocarcinoma	*FGFR2-BICC1*	*TSC1 p*.S410*, *BAP1 p*.E450fs*1	2	low*
5	Cholangiocarcinoma	*FGFR2-BICC1*	*MYC CNA(CN = 9)*	1	CBD
6	Colon Adenocarcinoma	*SUPT3H-ROS1*	*KRAS p*.*Q61H*, *APC p*.E1379*(LOH), *TP53 p*.*R282W(LOH)*	3	low
7	Glioblastoma multiforme	*FGFR1-TACC1*	*-*	0	low
8	Low-grade glioma	*KIAA1549-BRAF*	*-*	0	low
9	Neuroendocrine tumor	*SORCS1-RET*	*CDK6 CNA(CN = 98)*	1	low*
10	Pancreatic Adenocarcinoma	*PPP1R9A-BRAF*	*BAP1 p*.Q261*	1	low
11	Prostate adenocarcinoma	*SPINT1-IGF1R*	*MYC CNA(CN = 11)*, *RB1 HD*, *TP53 p*.*K132E(LOH)*	3	low
12	Skin cutaneous melanoma	*SUGT1P1-FGFR1*	*CDKN2A HD*, *CDKN2B HD*, *MTAP HD*	3	low
13	Stomach adenocarcinoma	*FGFR2-TACC2*	*MAP3K13 p*.*I523V(LOH)*, *TP53 p*.C229fs*10	2	low

AA, Actionable alterations; TMB, tumor mutation burden; LOH, loss of heterozygosity; CNA, copy number alteration; CN, copy number; HD, homozygous deletion; CBD, cannot be determined; TMB-low, TMB <10 Muts/Mb.

*Blood TMB analyzed using blood-based comprehensive genome sequencing.

We identified multiple complex chromosomal rearrangements involving *NUTM1*. These included a possible translocation between *NUTM1* on chromosome 15q and a region on chromosome 19p upstream of *BRD4* and a possible *BRD4-NUTM1* fusion in two patients (one with NUT carcinoma and another with porocarcinoma) (Table 2). However, whether this fusion observed in porocarcinoma leads to the BRD4-NUTM1 fusion, which is characteristic of NUT carcinoma, remains unknown.

### Cancers with kinase fusion tended to have fewer actionable alterations

**Fig 2:**
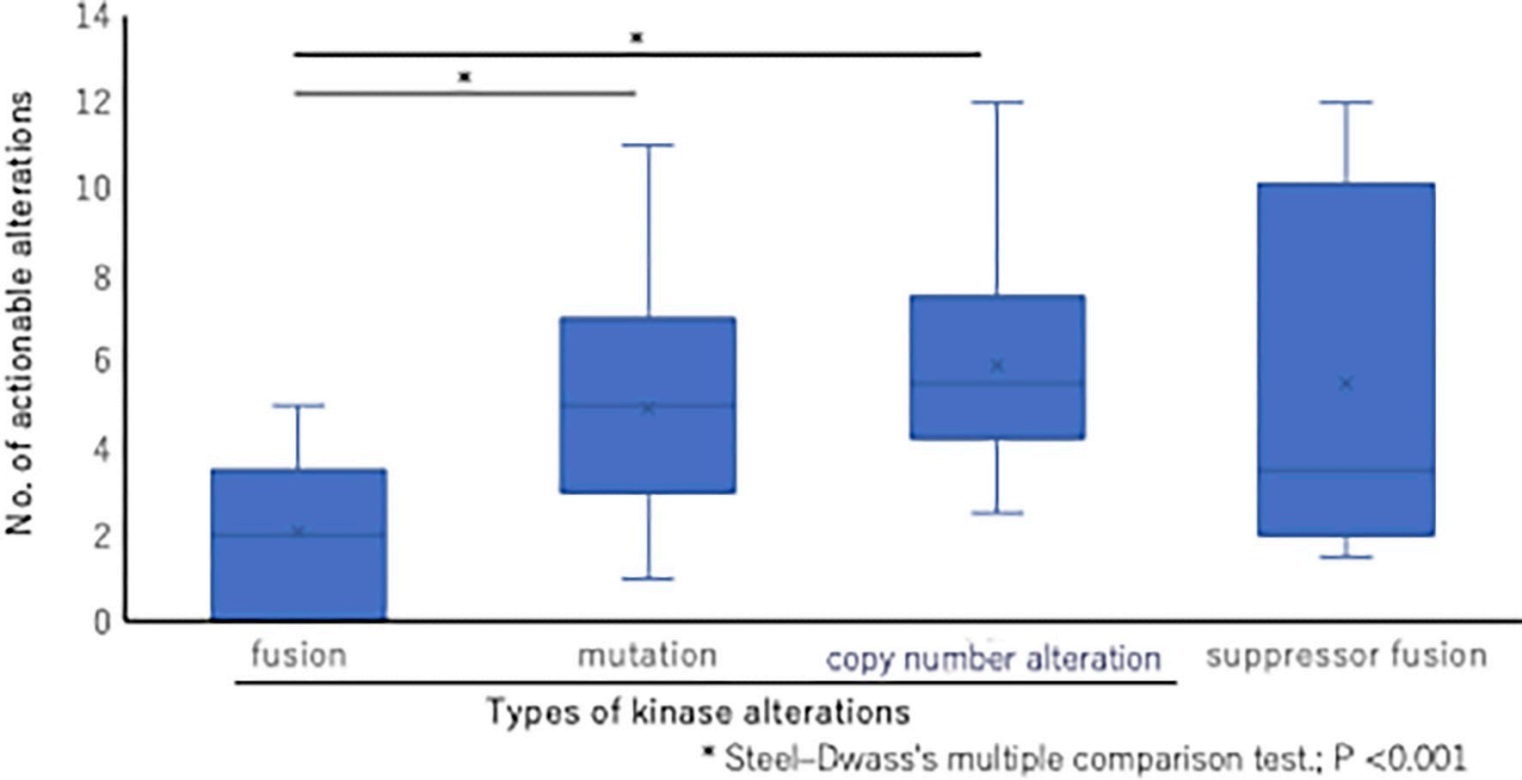
Distribution of actionable alterations across each alteration group. Patients with kinase fusions had significantly fewer actionable alterations than patients with kinase mutations and CNAs (*P* < 0.001; Steel–Dwass multiple comparison test). However, fusions involving tumor suppressors other than kinase fusion did not significantly differ in the number of actionable alterations.

We identified 117 patients (19.1%) with mutations and 58 patients (9.5%) with CNAs involving the kinase domain (Table 1). After adjusting for baseline characteristics (sex, age, smoking, heavy drinking, prior chemotherapy, and previous targeted therapy), we applied comprehensive genomic testing using F1CDx or F1LCDx to detect the frequency of actionable alterations in patients with kinase-domain fusions (n = 13), mutations (117), CNAs (n = 58), and suppressor fusion (n = 9). The median counts (interquartile range [IQR]) were 2 (1–3), 5 (3–7), 6 (4–8), and 4 (2–9), respectively (*P* < 0.001). The number of alterations was significantly lower in patients with kinase fusion than in those with kinase-domain mutations or CNAs (*P* <0.001; Steel–Dwass multiple comparison test) (Fig 2). However, the number of alterations was not statistically different for patients with suppressor fusions compared with that for those with kinase-domain mutations and CNAs (Fig 2). Although there were no statistically significant differences, only one of the 13 patients with fusion aberrations was found to be a heavy drinker (Table 1). In addition, the cancers with transcriptional-regulator fusion tended to have fewer actionable alterations 2 (0–3.5) (median [IQR]). In addition, TMB was low in all but one of the 13 patients with kinase-domain fusions that could not be analyzed (Table 3).

## Discussion

These findings reveal that 7.5% of the 613 patients exhibiting 40 types of solid cancer included in the cohort had tumors with fusions. Notably, kinase fusions, which may have particular structural properties selected during oncogenesis, were detected in 2.6% of the patients, accounting for nine solid-cancer types. Cancers with kinase fusions tended to have fewer actionable alterations than those with kinase mutations and CNAs. Furthermore, the other kinase-domain mutations and CNAs were generally mutually exclusive in most patients with kinase fusions. These findings suggest that kinase fusion is a strong biological driver of cancer development. It is well established that CGS of tumor DNA is less sensitive than other methods for detecting fusions. This is a limitation of the study and requires further elaboration. Furthermore, some tumors with fusions may have been missed, and data from those cases may have been included in other cohorts (for example, cases of kinase mutation only). Detection of fusion in DNA-sequencing data is difficult, and bimodal DNA- and RNA-based gene panels can be useful for this detection.

The presence of *IGF1R-SPINT1* in patients with prostate adenocarcinoma, which we detected in patient 8, has not been previously reported. This fusion protein lacks an extracellular domain but retains the kinase domain. Therefore, removing the entire extracellular domain of the insulin-like growth factor receptor activates the receptor, even without bound insulin-like growth factor [11]. Although, we did not perform any cell culture-based analysis to demonstrate that this fusion has transformation ability, our results as well as those of some of the previous studies suggest that the *IGF1R-SPINT1* fusion represents an activating mutation.

In patients 5, 6, 9, 11, and 13, we detected the coexistence of oncogene alterations with kinase fusions, considered generally mutually exclusive (Table 3). The CNA of *MYC* in patients 5 and 11 was limited to a few copies: CN = 9 and CN = 11, respectively. Furthermore, since the TSG (such as *RAD21* or *NBN*) in chromosome 8q, where *MYC* is present, was amplified with the same copy number, it was considered to be the result of polysomy of chromosome 8q. In addition, *KRAS p*.*Q61H* in patient 6 was interpreted as a conflicting interpretation of pathogenicity in ClinVar. *MAP3K13* (encoding LZK)-amplified head and neck squamous cell carcinoma cells harboring 3q gain depend on LZK expression for cell viability and colony formation [16]. Notably, *LZK* expression is required for cell proliferation and anchorage-independent growth in *MYC*-overexpressing breast and hepatocarcinoma cell lines [17]. However, alterations that are predicted to inactivate LZK have also been reported in breast cancer [18]. Therefore, whether *MAP3K13 p*.*I523V*(LOH) in patient 13 functions as a tumor suppressor or an oncogene remains unclear. In these patients, the kinase fusions appear to be critical for tumorigenesis, even with the coexistence of the oncogene alterations. There is only one coexisting actionable alteration; however, a high CNA of *CDK6 (CN = 98)* was detected in patient 9. Sitthideatphaiboon et al. defined the pathways limiting EGFR-inhibitor response, including the cell-cycle-gene, *CDK4/6* CNA [19]. In such patients, the effect of TKIs may be influenced by the number and type of comorbid actionable alterations. Notably, in all 13 patients, tyrosine kinase fusions were mutually exclusive of other tyrosine kinase domain alterations. These results indicate that tyrosine kinase fusions are pivotal in tumorigenesis and are most likely effective targets for molecular therapeutics.

Molecularly targeted drugs have been most successful in treating chronic myelogenous leukemia, driven by a kinase fusion (*BCR–ABL1*) [4]. Integrative genomic analysis revealed cancer-associated mutations in only three out of 19 patients (16%) who responded optimally to imatinib, whereas cancer-gene variants were detected in 15 of 27 patients (56%) with poor outcomes [20]. In our study, sole kinase fusion, without other actionable alterations, may significantly influence cancer development in three patients (with cholangiocarcinoma, glioblastoma multiforme, and brain low-grade glioma). However, in three patients with lung adenocarcinoma harboring an activating EGFR kinase mutation (EGFR p.E746_A750del, p.S768I, and p.L858R) without previous EGFR-targeted therapies, we detected seven, four, and three actionable alterations, respectively. Moreover, all three patients had *TP53* loss-of-function mutations in uniparental disomy (S1 Table). *TP53* mutations are associated with faster resistance evolution in EGFR-mutant NSCLC and mediate the acquisition of resistance mutations to EGFR TKIs [21]. Therefore, it is critical to validate tumor alterations, including driver and actionable mutations using CGS, to evaluate the efficacy of molecularly targeted therapy.

In our study, patients with kinase fusions tended to drink less. Reportedly, among Japanese patients, the gene mutation mechanism is strongly associated with drinking; moreover, single base substitution of *TP53*, *CDKN2A*, *PIK3CA*, *NFE2L2*, and *NOTCH1* induces esophageal squamous cell carcinoma [22]. The lack of actionable alterations in cancers with fusion is thought to be consistent with the fact that these cancer types are not associated with alcohol consumption. Moreover, the TMB was low in all of the 13 patients with kinase-domain fusion except in one patient in whom it could not be analyzed. Focusing on kinase fusion, Gao et al. [14] showed that many patients harboring fusions involving cancer-driver genes had no driver-gene mutations. Moreover, their analysis highlights an important consideration for immunotherapy in patients with fusions. Specifically, the significantly lower mutational burden observed in patients with driver-gene fusions points toward a reduced efficacy of immunotherapy in these patients despite fusion peptides being potentially good immunogenic targets. Based on their findings, Gao et al. [14] suggested that further research into driver-gene fusion can result in the development of targeted drugs and immunotherapy.

This study’s small sample size is a limitation of this study. We only included 13 patients with kinase-fusion in the analysis; therefore, the analysis may not have been sufficiently powered to derive conclusions. Therefore, future analyses using larger sample sizes are necessary.

## Conclusion

Our results demonstrated that cancers with kinase fusion tend to have fewer actionable alterations than those with kinase mutations and CNAs, reflecting the strong dependence of tumorigenesis on kinase fusion. Therefore, detecting kinase mutations is crucial in developing molecularly targeted therapeutics and immune therapy.

## Supporting information

S1 Table Lung adenocarcinoma with *EGFR* mutation status. (DOCX)
