# Supplementary material for: Comparison of actionable alterations in cancers with kinase fusion, mutation, and copy number alteration
Source: PLoS One. 2025 Jan 23;20(1):e0305025. doi: 10.1371/journal.pone.0305025 (PMC11756797; doi:10.1371/journal.pone.0305025)
Supplement: S1 Table — (DOCX) [file pone.0305025.s001.docx]

S1 Table. Lung adenocarcinoma with *EGFR* mutation status

| **Patient** | ***EGFR* mutation** | **AA** | **No. of AA** |
| --- | --- | --- | --- |
| 1 | *p.E746_A750del* | *RB1 HD*  *ASXL1 p.P1272A(UPD/LOH)*  *RAC1 CNA(CN=6)*  *CARD11 CNA (CN=6)*  *TP53 p.V97fs*25(UPD)*  *MUTYH c.892-2A>G*  *CDK8 CNA(CN=8)* | **7** |
| 2 | *p.S768I* | *SMAD4 p.Q245*(UPD)*  *MLL2 p.V1561G(UPD)*  *APC p.A1457fs*16(UPD)*  *TP53 p.E224*(PLS=3; UPD)* | 4 |
| 3 | *p.L858R* | *RBM10 p.Y582**  *TP53 p.S241F(UPD)*  *RB1 p.W99*(UPD)* | 3 |

AA, Actionable alterations; UPD, uniparental disomy; LOH, loss of heterozygosity; CNA, copy number alteration; CN, copy number; HD, homozygous deletion; EGFR, epidermal growth factor receptor.
